# Pulse Rate Measurement During Sleep Using Wearable Sensors, and its Correlation with the Menstrual Cycle Phases, A Prospective Observational Study

**DOI:** 10.1038/s41598-017-01433-9

**Published:** 2017-05-02

**Authors:** Mohaned Shilaih, Valérie de Clerck, Lisa Falco, Florian Kübler, Brigitte Leeners

**Affiliations:** 1 Department of Reproductive Endocrinology, University Hospital Zurich, Zurich, Switzerland; 2 Ava AG, Zurich, Switzerland

## Abstract

An affordable, user-friendly fertility-monitoring tool remains an unmet need. We examine in this study the correlation between pulse rate (PR) and the menstrual phases using wrist-worn PR sensors. 91 healthy, non-pregnant women, between 22–42 years old, were recruited for a prospective-observational clinical trial. Participants measured PR during sleep using wrist-worn bracelets with photoplethysmographic sensors. Ovulation day was estimated with “Clearblue Digital-Ovulation-urine test”. Potential behavioral and nutritional confounders were collected daily. 274 ovulatory cycles were recorded from 91 eligible women, with a mean cycle length of 27.3 days (±2.7). We observed a significant increase in PR during the fertile window compared to the menstrual phase (2.1 beat-per-minute, p < 0.01). Moreover, PR during the mid-luteal phase was also significantly elevated compared to the fertile window (1.8 beat-per-minute, p < 0.01), and the menstrual phase (3.8 beat-per-minute, p < 0.01). PR increase in the ovulatory and mid-luteal phase was robust to adjustment for the collected confounders. There is a significant increase of the fertile-window PR (collected during sleep) compared to the menstrual phase. The aforementioned association was robust to the inter- and intra-person variability of menstrual-cycle length, behavioral, and nutritional profiles. Hence, PR monitoring using wearable sensors could be used as one parameter within a multi-parameter fertility awareness-based method.

## Introduction

Despite years of progress in fertility research, a user-friendly, non-invasive, and affordable method to estimate the female fertile window remains elusive. While calendar based methods offer an appealing solution their accuracy is questionable^1^. In addition, the considerable inter- and intra-person cycle variability limits their utility for a large fraction of the population^2^, especially given the frequent occurrence of anovulatory cycles (12–37%)^3, 4^. Therefore, methods that rely on physiological parameters reflecting the fertile window represent a more consistent predictor.

It is well-known that levels of estrogen and progesterone change with the different phases of the menstrual cycle^3^. In addition, both hormones are known to affect the cardiovascular system through various mechanisms. Furthermore, these effects are mirrored on the heart rate throughout the different menstrual phases^5^. Moran *et al*.^5^ demonstrated that pulse rate significantly increases during the fertile window compared to the menstrual phase (fertility was established by directly measuring reproductive hormones using first morning urine). The authors of the study acknowledged the difficulty associated with the study protocol given the need for participants to be present in the clinic daily. Photoplethysmography (PPG) technology is a noninvasive, affordable, and user-friendly method to measure pulse rate. In addition, wearable PPG technology has been established to be an accurate estimation of PR in healthy individuals during rest and sleep^6^. In contrast to clinical settings, wearable sensors allow for the continuous monitoring of physiological parameters under representative normal living conditions, including sleep.

Sleep is considered a more favorable condition to measure the influence of sex hormones on cardiac activity, as it minimizes the influence of external conditions that can affect the heart rate^6–8^. Only a few studies have investigated the effect of menstrual cycle phase on PR during sleep; they all reported an increase during the luteal phase^9–11^.

Earlier studies that took place during daytime demonstrated the association of PR and the different phases of the menstrual cycle. One study reported an increase in PR at ovulation^12^, and several found an increase during the luteal phase^13–15^. Other studies did not find a significant difference in daytime resting PR between different phases of the menstrual cycle during the day^16–22^. The aforementioned studies used a heterogeneous set of methodologies, and measured PR during the day, hence the measurements were probably susceptible to various confounders, which could explain the discrepancy observed.

None of the earlier studies concerning the menstrual cycle phase and pulse/heart rate utilized wearable sensors technology, and the measurements were all conducted in clinics, which does not reflect the normal conditions for the majority of individuals interested in fertility monitoring. The aim of this study is to investigate the pattern of PR throughout the menstrual cycle when measured at home during sleep using wearable PPG sensors. We also aim to assess whether the changes in pulse rate throughout the menstrual cycle correlate with the fertile window. Given the aforementioned effects of the different reproductive hormones on the cardiovascular system, pulse rate could potentially be used as a predictor of reproductive hormone levels throughout the menstrual cycle, and assist in identifying the fertile window.

## Results

### The study population

A total of 91 participants conformed to the study inclusion criteria (see methods). The participants were on average 33.2 years old (±4.7), with an average height of 168.3 centimeters (±6.1), and an average weight of 61.4 kilograms (±9.3), resulting in a mean body-mass index of 21.6 kilogram/meter^2^ (±2.8).

Of the 91 participants, 58 wore device 1 and logged 224 cycles, 26 wore device 2 and recorded 132 cycles, while 7 women wore device 3 and recorded 23 cycles, for a total of 379 recorded cycles. Overall, 105 cycles (27%) were excluded, 99 (26%) due to unconfirmed ovulation using the urine fertility test, and 6 (2%) others due to participant mal-adherence to the study protocol. The average number of recorded nights per cycle was 22.3 days, with the mean ovulatory cycle length being 27.3 days (±2.7). The average number of cycles recorded per subject was 3.3 (±1.5). Despite the participants reporting regular cycle length at recruitment (24–35 days), 56 (15%) of the cycles collected during the study were below or above that range.

### Pulse rate correlation with the different phases of the menstrual cycle

**Figure 1:**
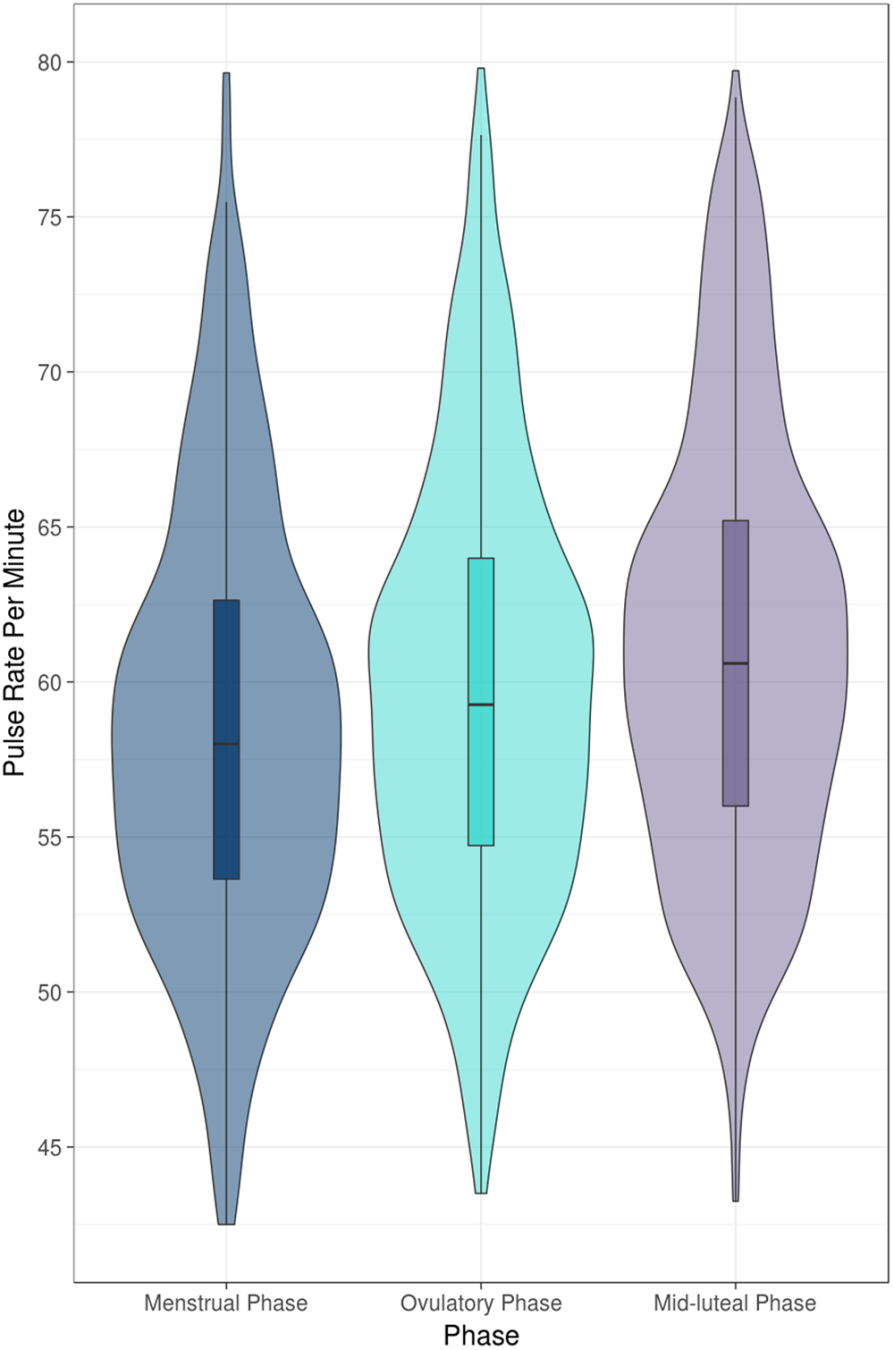
Violin plot of the pulse rate in the different phases of the menstrual cycle.

We found a significant increase in the median pulse rate during the fertile window (OV-5 to OV) compared to the menstrual phase (estimated change 2.1 beat-per-minute (BPM), standard error (SE) 0.2, *p* < 0.01) (Table 1, Fig. 1). Furthermore, the median pulse rate during the mid-luteal phase (OV + 3 to OV + 9) was also significantly higher than during the fertile window (1.8 BPM, SE 0.1, *p* < 0.01), and the menstrual phase (3.8 BPM, SE 0.2, *p* < 0.01).

**Table 1:** Uni- and multi-variable mixed effects model of pulse rate and the various physiological phases and the potential confounders (showing estimated change in PR per covariate).

Covariate	Univariable model, estimated change in BPM (SE)	Multivariable model^#^, estimated change in BPM (SE)
Menstrual phase		
Menstruation	Reference	Reference
Ovulatory phase	2.09 (0.2)***	2.19 (0.20)***
Mid-luteal phase	3.81 (0.19)***	4.05 (0.20)***
Alcohol^§^		
No alcohol	Reference	Reference
1–4 units	1.56 (0.12)***	2.11 (0.21)***
5 or more units	5.90 (0.31)***	8.27 (0.53)***
Large meal^§^ (binary)	1.56 (0.18)***	0.71 (0.28)*
Exercise^§^		
No exercise	Reference	Reference
<60 minutes	−0.21 (0.16)	0.05 (0.24)
> = 60 minutes	0.48 (0.21)*	0.48 (0.30)
Body mass index (KG/M^2^)	0.70 (0.24)**	0.65 (0.24)**
Weight (KG)^a^	0.16 (0.08)*	—
Sex^§^ (binary)	0.10 (0.21)	—
Shower^§^,^▫^ (binary)	0.30 (0.22)	—
Age (year)	−0.09 (0.17)	—
Height (centimeter)	−0.12 (0.13)	—
Coffee	0.36 (0.20)	—

^#^Covariates not-included in the multivariable model are shown with a “—” in the table. ^§^Within a four-hour period before sleep. ^▫^Data available for participants with device 2 and 3 only. ^a^Given the collinearity between weight and body mass index, only body mass index was included in the multivariable model. *, **, *** refer to a p-value < 0.05, 0.01, 0.001, respectively.

We then analyzed irregular cycles (56 cycles) separately to assess whether the same associations could be observed. Indeed, we observed the same significant increase of PR between the fertile window and the menstrual phase, however the magnitude of the increase was marginally smaller (1.8 BPM, SE 0.35, p < 0.01). The increase of PR between the mid-luteal phase and the menstrual phase was also minimally reduced (3.2 BPM, SE 0.35, p < 0.01).

The correlations between pulse rate and the menstrual phases were robust to adjustment for the effect of confounders in a multivariable model (Table 1). The multivariable model also showed that the baseline pulse rate within participant had a standard deviation (SD) of 6.9 BPM, and within cycle the SD was 1.6 BPM. In addition, the definition of the mid-luteal phase (varying start OV + 2–7 and varying end OV + 9–11) did not qualitatively change the correlation (data not shown).

### Effect of confounders on pulse rate

We observed in the univariable models that the consumption of alcohol, large meals, or intense exercise (> = 60 minutes) within the 4 hours preceding sleep significantly increased median pulse rate during the night (Table 1). In contrast, having a shower before sleeping, drinking more than 200 mg of caffeine, and exercise of less than 60 minutes within 4 hours before sleep did not have a significant effect on the median pulse rate (Table 1). The correlations (or the lack thereof) between the confounders and pulse rate were not dependent on the menstrual phase when an interaction term between the respective confounder and the cycle phase was included in the model (each in a separate model, data not shown). Finally, weight and BMI were associated with an increase in the median resting pulse rate measured through the night, while height was not (Table 1).

## Discussion

We demonstrated in this study that the median pulse rate measured through wrist worn wearable sensors strongly correlates with the different phases of the menstrual cycle. More specifically, PR during the fertile window was significantly increased compared with the menstrual phase, with this increase carrying through the luteal phase to reach a PR peak during the mid-luteal phase. Our study also demonstrates the robustness of PR correlation with the menstrual phase given a broad spectrum of menstrual cycle duration, under a range of different daily activities such as the consumption of coffee, alcohol, and large meals before sleep as well as other activities such as sports, showers, and intercourse or on the background of different BMIs.

Our results are consistent with previously reported patterns of resting pulse rate measured through the menstrual cycle in clinically controlled environment^5^. This suggests that wearable PPG sensors could be a reliable convenient method to assess the current phase of the menstrual cycle through PR measurement. The mechanisms by which heart rate is increased in the different phases is thoroughly discussed by Moran *et al*.^5^. Novel in our study is the demonstration that the association between the elevated pulse rate of the ovulatory phase compared to menstrual phase is observable even in irregular cycle lengths. Individuals with highly variable cycle length represent one of the populations that could benefit most from our proposed method given that a calendar based method would be ineffective in their case, and urine based testing would require a much longer phase of daily urine testing. The magnitude of the association was different between individuals with regular and irregular cycle length. However, this difference could be attributable to the smaller number of irregular cycles at hand (56 cycles).

Given our findings, pulse rate is a strong candidate for inclusion in modern multi-parameter fertility awareness based methods (FABM). To date, cyclic changes in cervical mucus and basal body temperature have been the two most commonly used physiological parameters to estimate the timing of the fertile window^23, 24^. However the basal body temperature method is a poor predictor of ovulation and requires strict measurement protocols^25^. The self-detection of peak mucus day, defined as clear, slippery and lubricative mucus, is a better predictor of ovulation and is reported to fall within the fertile window in 72% of the cycles in which ovulation was predicted with LH-urine tests^26^. However its utility is highly dependent on the subjective interpretation of the user and requires both experience and a certain level of comfort with the procedure^27^. A combination of these natural fertility markers and the calendar method, optionally together with the use of electronic and chemical fertility devices, are becoming more popular as FABMs to achieve or avoid pregnancy^28, 29^. The main advantage of modern FABMs is the lack of medical side effects compared to hormonal contraception and a higher accuracy compared to single indicator methods. The use of wearables might help to overcome disadvantages of present FABMs such as inconvenient time-consuming measurements, limited robustness because of isolated, eventually non-representative, data points, difficulties with the interpretation of findings etc. For example, the bracelets used in the present study allow convenient measurements of every pulse in t a night (24 000 beats, for 8 hours of sleep, and an average PR of 50 BPM). When used correctly, today’s FABMs, combined with electronic and chemical fertility devices, have similar unintended pregnancy rates compared with conventional methods (e.g. condoms)^23, 29–31^. Future studies will have to show whether FABMs completely relying on wearable sensor measurements could allow further convenience with comparable efficacy.

As with all observational studies there are limitations to ours. A fraction of the cycles was excluded due to the lack of peak fertility as determined by the home administered urine test. Yet the rate we observed was within the expected range^4^. Even though the LH-urine tests are considered a reliable predictor of ovulation^32^, Behre *et al*.^33^ showed that only in 76.2% of the cycles urine based test showed that ovulation occurred on the second day of peak fertility. In addition, one cannot exclude administration and equipment malfunctions. More accurate determinants of the ovulation day (e.g. using ultrasound, lab measured reproduction hormones from the serum, saliva, or urine) would probably result in a more accurate estimate of the magnitude of the association. The study population was largely of Caucasian ethnicity with normal BMIs. Further studies with more physiologically and ethnically diverse population are required to assert the generalizability of the findings. In this study, three different wearable sensor bracelets were deployed, yet no difference was observed in the associations given the different sensors in either the univariable or the multivariable analysis.

In conclusion, we observe a significant increase in median pulse rate during the fertile window (OV − 5 to OV) compared to the menstrual phase, with the median pulse rate sustaining this increase to reach a maximum during the mid-luteal phase. The associations were present in cycles with varying lengths, and under a broad range of daily activities and nutrition profiles. These findings indicate that pulse rate (measured during sleep) is a promising parameter for FABMs. And PR monitoring using wearable PPG sensors could be used as a conveniently measured parameter within a modern multi-parameter fertility awareness-based method.

## Material and Methods

### Participants

Volunteers were recruited via the Department of Reproductive Endocrinology, University Hospital Zurich, but were not in treatment. The inclusion criteria were an age between 18–42 and a self-reported regular cycle in the six months preceding the study. In addition, none of the subjects used any kind of reproductive hormone therapy three months before and during the study period. Study participants were screened prior to the study to exclude endocrine pathologies. This consisted of the patient’s history, a general and a gynecological exam including a transvaginal ultrasound. Moreover, thyroid stimulating hormone, prolactin, estradiol and follicle stimulating hormone levels were evaluated in the early follicular phase (cycle days 2–5). Women with health conditions or women taking medication or other substances that may affect the menstrual cycle, or the physiological parameters investigated were excluded from the study. Other exclusion criteria were frequent travel between different time zones and sleep disorders. Premature withdrawal criteria were: pregnancy, health issues, non-compliance with the study protocol, and a participant’s choice to withdraw.

### Study design

The data was collected from two clinical trials, both of which were a single-center prospective observational trial conducted at the Department of Reproductive Endocrinology, University Hospital Zurich. All participants provided signed informed consent, and the study was approved by the Ethical Commission of the Canton of Zurich (approval number: KEK-ZH-Nr. 2015-0018). The study has been conducted according to the relevant rules and regulation. The total study period of the first clinical trial was 1 year and each subject was intended to participate for 6 months. The duration of the second clinical trial is 18 months.

### Study protocol

Each of the subjects wore one of 3 CE-certified wearable devices at night during sleep under normal living conditions. All devices contain a PPG sensor to record pulse rate. The bracelet was placed on the dorsal surface of the wrist. Moreover, the participants were required to wear the bracelet on the same arm during the study period, however, the choice of arm was free.

A self-administered home-urinary test (Clearblue Digital Ovulation Test) was used to estimate the ovulation day. Said test has been demonstrated to be a good estimator of ovulation^33, 34^. The test detects estrone-3-glucuronide and luteinizing hormone (LH) concentrations in the first morning urine. The instructions provided in the package insert were used to determine when the participant was to start with the tests during the pre-ovulatory period. When the test detects a significant rise in estrogen levels above the individual baseline levels, it shows a blinking smiley, indicating ‘high fertility’ days. Once LH exceeds a certain threshold level, a constant smiley, indicating ‘peak fertility’ days is displayed for 48 hours. The estimated day of ovulation was then considered as the first day of ‘peak fertility’ plus 1 day. Instructions for use of the device were provided to participants in detail by a study nurse, and all subjects were asked to use the test according to the package insert instructions, with one exception. That is taking the first morning urine.

Participants were asked to complete a daily electronic survey throughout the study period. The questionnaire included questions regarding the menstrual phase, onset and duration of menses, day of a positive LH-test, sleeping habits, sleep quality, mood, stressful private and/or professional events, alcohol (1 unit of alcohol = 10 g pure alcohol: 4 centiliter schnapps, 1 deciliter wine/champagne; 2 deciliters long drink; 2.5 deciliter beer), caffeine consumption, heavy meals (>600 kilocalorie), and work outs and their intensity. The collected survey covariates are known to affect heart rate and hence were collected to correct for their potential confounding roles^35–37^.

### Data collection and processing

Photoplethysmography (PPG) technology was used to measure pulse rate. Three wrist worn bracelets were used, the first and second devices (Ava bracelet, Ava AG, Switzerland; and PulseOn bracelet) contains a 2-wavelength optical PPG sensor. The third device (Basis Peak, BASIS Science Inc) contains a 1-wavelength optical PPG sensor. All devices were demonstrated to have good correlation with heart rate collected using hospital grade electrocardiogram^38, 39^.

Collected pulse rate data from the individual devices were filtered with a 5 minutes median filter. The first 4 hours of sleep and the last 30 minutes were removed from the recordings of every night to allow for PR to stabilize (as the second and third bracelets used did not provide sleep classification hence we could not filter the data based on the sleeping phase). Pulse rate per night was computed as the median of the remaining data.

The relevant phases of the menstrual cycle were defined for analysis into 3 phases: the menstrual phase (days 1 to 5), the fertile window (OV − 5 to OV) and the mid-luteal phase (OV + 3 to OV + 9). As a sensitivity analysis, we assessed whether different definitions of the mid-luteal phase have an impact on the association. The alternative definitions were varying the beginning of the mid luteal phase between varying OV + 2–7, and varying the end between OV + 9–11.

### Statistical analysis

The results were calculated using R (with the following packages^40–44^) and Matlab (Mathworks, Inc.), and they are presented as mean (±SD) for the descriptive statistics, and for the modeled estimates as estimate (standard error (SE)). Only complete cases were included in the analysis and statistical significance was set at a *P-value* < 0.05. Given the known intra- and inter-personal variance of the pulse rate we opted for a linear mixed effects model, and a random intercept per subject, and per cycle per subject. Such a model allows for the baseline pulse rate to change per person, and within the person said baseline pulse rate could change per cycle^45^. Pulse rate was the outcome variable, and the menstrual phases as well as other potential confounders were the explanatory variables. The confounders were assessed independently in univariable models, and when the P-value was <0.1 they were included in the final multivariable model.
